# Factors Associated with Visceral Leishmaniasis in the Americas: A Systematic Review and Meta-Analysis

**DOI:** 10.1371/journal.pntd.0002182

**Published:** 2013-04-25

**Authors:** Vinícius Silva Belo, Guilherme Loureiro Werneck, David Soeiro Barbosa, Taynãna César Simões, Bruno Warlley Leandro Nascimento, Eduardo Sérgio da Silva, Claudio José Struchiner

**Affiliations:** 1 Departamento de Endemias Samuel Pessoa, Escola Nacional de Saúde Pública Sergio Arouca, Fundação Oswaldo Cruz. Rio de Janeiro, Rio de Janeiro, Brazil; 2 Departamento de Epidemiologia, Instituto de Medicina Social, Universidade do Estado do Rio de Janeiro, Rio de Janeiro, Brazil; 3 Departamento de Epidemiologia e Métodos Quantitativos em Saúde, Escola Nacional de Saúde Pública Sergio Arouca, Fundação Oswaldo Cruz. Rio de Janeiro, Rio de Janeiro, Brazil; 4 Centro de Referência Nacional e Internacional Para Flebotomíneos, Centro de Pesquisas René Rachou, Fundação Oswaldo Cruz (FIOCRUZ), Belo Horizonte, Minas Gerais, Brazil; 5 Campus Centro-Oeste Dona Lindu, Universidade Federal de São João del Rei, Divinópolis, Minas Gerais, Brasil; RTI International, United States of America

## Abstract

**Background:**

Still today, more than 30 years after the beginning of the process of visceral leishmaniasis' urbanization, there is little knowledge about the risk factors for its occurrence, despite their relevance to the control and understanding of disease dynamics. The present study is the first systematic review with meta-analysis about factors associated with *Leishmania infantum* infection in humans in the Americas.

**Methods and Findings:**

After searching different databases, consultations to the reference lists of articles and to experts in the field, 51 studies were reviewed. Theoretical discussions or meta-analysis of p-values or of effect sizes were used to pool information about each variable. The Q test and the I^2^ statistic were used to assess heterogeneities among the studies. Male sex was associated with visceral leishmaniasis in studies which used the leishmanin skin test for diagnosis and in those where the outcome was the clinical disease; the opposite occurred when serological diagnosis was applied. Younger individuals were less frequently infected than adults, but were more prone to illness. Although with different levels of evidence and of heterogeneity, the presence of dogs at home, higher dog seropositivity in nearby areas, lower socioeconomic status and highly vegetated areas were associated with *L. infantum* infection. This was not noticed for the presence of chickens in the house and with nutritional status. Susceptibilities to bias and limitations in the analysis and in the description of results were often identified in the studies analyzed.

**Conclusions:**

Results showed the existence of consistent patterns for some of the factors analyzed and should be taken into account in developing more effective and well-targeted control measures. Studies must be conducted in new areas of the continent, with improved methodological quality and prioritizing the investigation of the patterns identified and their causes, as well as variables for which knowledge is poor.

## Author Summary

Visceral leishmaniasis (VL) is the most severe clinical form of leishmaniasis. In the Americas, VL is a zoonosis caused by the protozoan parasite *Leishmania infantum*, an intracellular micro-organism transmitted to humans through the bite of female phlebotomine sand flies. The domestic dog is considered the main reservoir and human beings are dead-end hosts. The disease occurs in 12 countries of the New World and Brazil accounts for 90% of the reported cases. The factors associated with infection are only partially understood, making harder understanding the disease dynamics and the development of effective and well-targeted control measures. Our work is the first systematic review with meta-analysis about factors associated with human visceral leishmaniasis in the Americas. It provides an organization of the available information, the description of more accurate data on the role of each of the factors studied, the evaluation of possible causes of heterogeneity in the results of the studies as well as the analysis of the quality of publications and limitations in the existing knowledge. The results of this study might be useful in the definition of which and where target interventions should be delivered, and for guiding researchers towards the development of future studies of better methodological quality.

## Introduction

In the Americas, visceral leishmaniasis (VL) is a zoonosis caused by the protozoan parasite *Leishmania infantum* (syn. *L. chagasi*). The disease is transmitted to humans by the bite of female sandflies of the genus *Lutzomyia* [1]. *L. longipalpis* is the main vector species in the New World [2] but transmission can also occur through *Lu. cruzi* and *Lu. evansi* [3], [4]. Other forms of transmission have already been recorded: congenital, drug injection and blood transfusion [5], [6]. Potential wild disease reservoirs are the fox (*Lycalopex vetulus* and *Cerdocyon thous*) and the opossum (*Didelphis albiventris*) [7]. Dogs (*Canis familiaris*) are considered the main reservoir in the urban setting [8]. Clinical manifestations of the disease can range from asymptomatic forms to severe visceral involvement [9]. The incubation period lasts around two to six months [10]. The fatal outcome occurs predominantly by co-infection or bleeding complications [11]. Studies in Brazil show a variation in case-fatality rates between 4.2% and 10.2% in treated patients [12].

For the diagnosis of human visceral leishmaniasis, delayed hypersensitivity tests, such as the Montenegro skin test (leishmanin skin test) [13], parasitological, serological (e.g., the Indirect fluorescent antibody test - IFAT, the enzyme-linked immunosorbent assay - ELISA, the direct agglutination test - DAT) and molecular methods are commonly used [14]. Immunological techniques are more often used in epidemiological studies since they are easier to implement.

The burden of the disease is not exactly known in the Americas, since there is a lack of effective surveillance systems [15]. Although the Brazilian surveillance system is considered better than in other Latin American countries, VL is also underreported in this country [16]. Previously known as a rural endemic disease, visceral leishmaniasis has become endemic and epidemic in large Brazilian cities since the 1980s [17]. The country accounts for 90% of reported cases in the Americas and is the third largest VL focus globally [15]. In Brazil, the recommended control actions against the disease are the use of insecticides against phlebotomines and culling of seropositive dogs, both strategies with little evidence of effectiveness and operationally costly [18], together with the identification and early treatment of human cases [19]. However, they have not produced effective results, given that neither incidence nor fatality rates were reduced in recent years. Moreover, the disease has spread to all regions of the country, and continues to expand into formerly disease-free cities [20]. Identification and quantification of risk factors for VL, besides being useful to understand the determinants of infection acquisition and disease development, can help develop more effective and well-targeted control measures [21].

There are several factors which may be associated with *L. infantum* infection in humans and many are considered controversial or only partially understood [18]. In this context of heterogeneity in study results, careful evaluation of its potential causes may, in meta-analysis procedures, be more useful than the mechanical calculation of summary measures [22]. Therefore, when summarizing studies that have addressed factors associated with VL, issues such as the quality of studies, design used, methods of data analysis, control for confounders, the way results were reported, and diagnostic tests employed must be considered in order to obtain the best evidence available.

In a systematic review of risk factors for visceral leishmaniasis in South Asia [23], consistent patterns have been described for some variables, such as: spatial clustering of cases, conditions of households, areas of vegetation near homes and poverty. However, due to the peculiar characteristics of the disease in that context (the etiological agent is *Leishmania donovani*; vector is *Phlebotomus argentipes*; and infected humans are the only reservoir [23]), results cannot be used to make predictions about other contexts. Specifically with respect to human American visceral leishmaniasis, there is no literature record of systematic reviews and meta-analysis regarding factors associated with infection by *L. infantum*. Thus, through the analysis of observational studies that investigated such factors, this study aimed to obtain more accurate data about each of the factors studied as well as to analyze the quality of publications and to identify gaps in existing knowledge.

## Methods

### Eligibility Criteria

Epidemiological cross-sectional, cohort, case-control and ecological studies were included in the review. These should have described associations between socioeconomic, environmental, family and individual variables and the occurrence of any outcome related to the acquisition of infection by *L. infantum*. This outcome could be the infection itself (as measured primarily in the study, regardless of the diagnostic assay or of the presence or absence of symptoms in the subject), the identification of active patients who had manifested the clinical disease (in the case of case-control studies) or the notification of cases by health services (in the case of ecological studies using secondary data). There was no restriction for age, sex or language.

We excluded studies conducted before 1980 or with populations other than those of the American continent, as well as reviews and papers published in scientific conferences or that were purely descriptive with no possibility of obtaining measures of association. With respect to the variables, we excluded those which referred to: a) control actions (not the focus of this review); b) subject's genetic characteristics (more related to differences in clinical prognosis); c) for how long a subject had been living in a household (relevant only in local contexts); d) the categorization of the subject's domicile as urban or rural (due to the lack of standardization in this definition). We maintained, however, those related to the environmental characteristics surrounding the households. We also excluded studies and variables whenever what was being described in the text was impossible to understand or when there were inconsistencies in quantitative data presented or flaws that could invalidate the association described.

### Search and Extraction of Information

All search strategies were conducted independently by two researchers (Belo, VS & Barbosa, DS) between March and June 2011. The terms used and databases consulted are described in Text S1 in the order they were searched. In addition, Brazilian research experts were consulted and searches through the reference lists of each article selected at the initial stage were conducted.

All titles and abstracts of identified articles were analyzed, being initially excluded those deemed irrelevant. When the information provided was not sufficient for the decision, or when studies were considered relevant for at least one of the researchers, the full text was analyzed. At this stage, papers were selected to be part of the present review.

The extraction of information contained in the publications was conducted by a researcher (Belo, VS) and reviewed by all authors of this article. We contacted authors of primary studies to obtain data necessary to calculate measures of association (and possible inclusion in meta-analysis) when they were not described in the publications. Individual patient data were not requested.

### Methods for Assessing Risk of Bias in Primary Studies

To identify potential risks of bias in studies and limitations in data analysis and in the way results were reported we used the STROBE statement. It issues recommendations that have been developed to improve the presentation of results from observational studies [24] including several items related to the existence of susceptibility to bias [25]. Additionally, questions from the Newcastle-Ottawa Quality Assessment Scale [26] (for assessing quality in observational case-control and cohort studies) and the book of Fischer and Getis [27] (for assessing quality in ecological studies) were consulted.

We have not used scales that result in a numerical quality threshold because this procedure involves arbitrary weighting of items, some of which may not to be directly related to the study's validity [25]. Thus, we decided to approach the problem more generally, pointing out the major limitations and susceptibility to bias of the revised studies.

Study quality and susceptibility to bias were used to discuss the limitations of existing knowledge, without interference, however, in meta-analysis procedures. There was no exclusion of studies due to those factors.

### Analysis and Summary of Results

Each association between a given exposure variable and a specific outcome was considered a separate and independent meta-analysis. Results of primary studies - except ecological - were described by Odds ratios (OR) and confidence intervals (CI). In cases in which there was information about the probability of significance (p-value), the direction of the association and the sample size of the study, the OR (or correlation coefficient) was estimated by reverse computation.

To make decisions about whether to combine or not effect measures, the agreement among the issues added by each study and the differences on their characteristics were evaluated. Whenever a summary measure of effect was obtained, the random effects model was used to pool the data. Under this model it is allowed that the true effect size varies from study to study, thus the overall variability includes the within-studies variance as well as the estimate of the between-studies variance. However, in cases where the number of studies was less than four, due to the lack of precision of the measure of variability [28], we preferred to use the fixed effects model.

The Q-Statistical test was used to analyze the occurrence of heterogeneity in effect sizes across studies. The I^2^ statistic was calculated to determine what proportion of the variance observed represented a real dispersion in the effect sizes (variation between studies), i.e. not due to random error (intra-study variation).

Due to the sufficient number of studies and the significant heterogeneity among them, subgroup analyses were carried out for gender, age, presence of dogs in the household and presence of chickens/birds. The following groups were considered: type of study (i. cross-sectional; ii. cohort; iii. case-control), method for measuring the outcome (i. leishmanin skin test (LST); ii. serological; iii. serological and LST; iv. other; v. clinical case), age group of participants (i. children; ii. adults; iii, all ages) and adjustment for confounding (i. yes; ii. no). The Q test was used for comparison of effect sizes and of heterogeneity between subgroups. Those which explain heterogeneity best (higher between subgroups and lower within subgroups) were described. For variables on which subgroup analyses were performed, studies were combined only within the subgroups and an overall measure was not calculated.

With regard to studies in which more than one diagnostic method had been performed, results of serological tests (ELISA firstly) were kept in the meta-analysis and results of LST were excluded. However, in analyses where studies were grouped by the method of measuring the outcome, the result of each technique was maintained and used for analyzing the subgroup to which it belonged.

The weighted Z-method for combining p-values [29] was applied when the diversity of studies in terms of design, populations or other characteristics became inadequate to obtain a summary-measure based on effect sizes, provided that the question analyzed was similar.

We investigated the existence of publication bias among the studies selected for each targeted association (provided that the number of publications is large enough) using the funnel plot, the Egger statistical test and the statistic of “Durval and Tweedie's Trim and Fill”. The last procedure re-computes the effect size imputing studies until the funnel plot is symmetric about the new effect size. Thus the best estimate of the unbiased effect size is calculated [28].

Theoretical discussions analyzing not only statistical significance, but also the strength and the direction of associations were conducted for variables on which it was not possible to use the methods presented.

To perform the meta-analysis of p-values, we used the software Meta-P and for remaining analyzes we used the software CMA, version 2.0.057.

## Results

### Selection of Publications and Limitations in Studies Included

**Figure 1:**
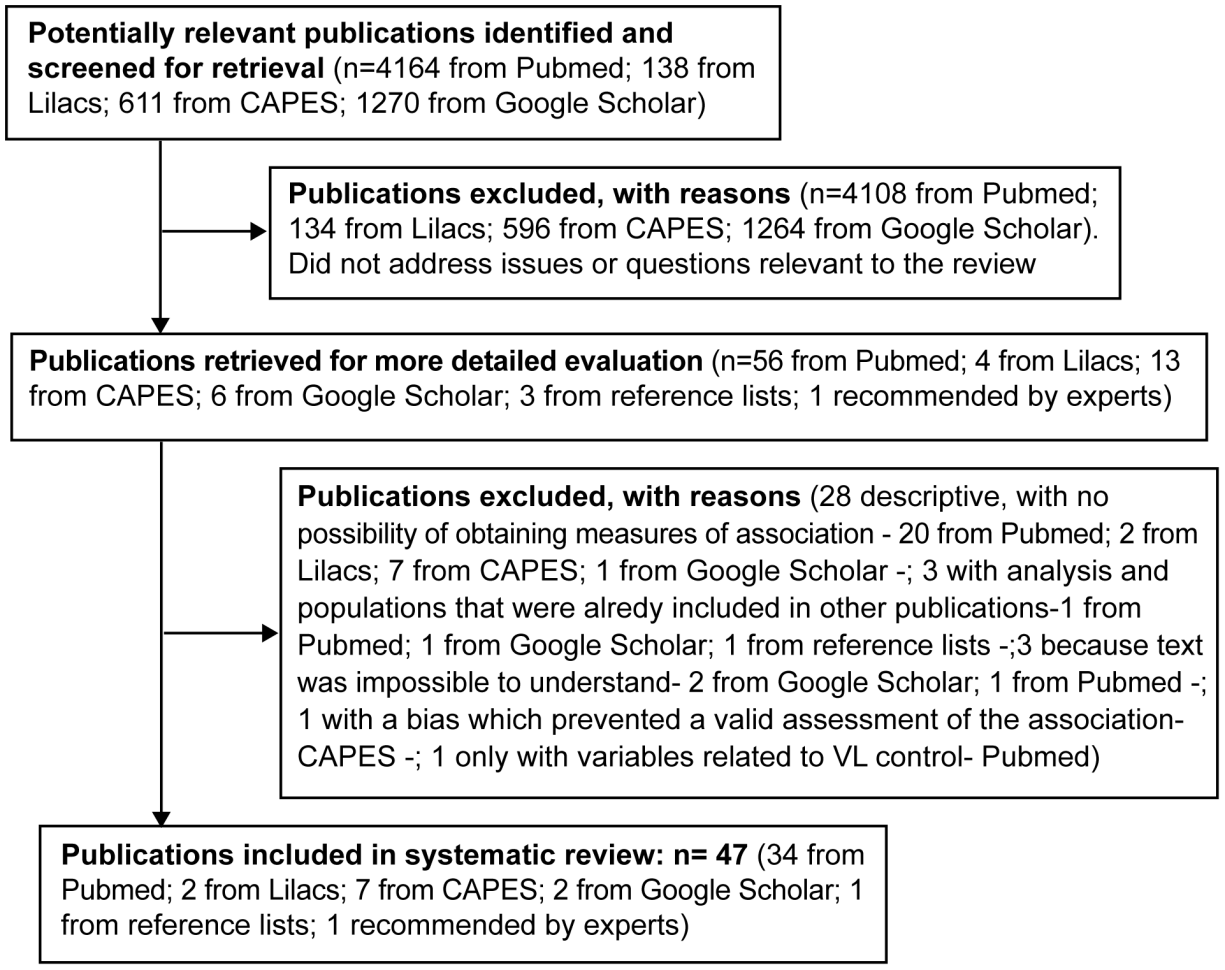
Flowchart showing the selection of studies analyzed in this review.

Forty-seven publications (51 studies) were included in the review. Figure 1 shows a flowchart of the search strategy and study selection, along with the reasons for exclusions.

Regarding the type of study, 24 were cross-sectional, 17 ecological, 7 case-control and 3 were cohorts. Three studies were conducted in Venezuela, two in Colombia, one in Honduras and forty-five in Brazil. In five studies the population consisted of children only. In studies where the infection was measured directly by investigators, the diagnostic test most often used was the LST, followed by ELISA and IFAT. In all case-control studies, cases were patients with clinical disease.

The main potential sources of bias in cross-sectional and cohort studies were the eligibility criteria employed and losses due to refusal to participate and during follow-up. In eight studies, the process of selection of participants was not clearly explained. In nine no reference was made to refusals or losses and in sixteen studies, although they have been quantified, there was no discussion about those profiles and the way they could have affected the results. With respect to case-control studies, there were also limitations on the description of the selection of participants and refusals in three studies. In four there was a clear possibility of recall bias. In ecological studies, in addition to limitations inherent to the design and use of secondary data, we detected the possibility of ecological fallacy in five of them and no verification of addresses of cases in six studies.

Regarding the limitations of the analyses, only thirteen studies did some kind of control of confounding. Except for two, none of the others described the criteria used for categorizing continuous variables. In ten the strength of the associations was not considered, and only statistical significance was analyzed. In six studies the normality of the outcome variable was not checked, although this was necessary for the statistical tests applied. Specifically for ecological studies, seven did not take into account the possible spatial dependence of data.

The main limitations of data presentation were related to the non-description of results for variables that were not statistically significant. In addition, several variables had to be excluded because it was not possible to understand the text description or the analyses performed.

In general, there were no differences in the quality of published as compared to unpublished studies. The limitations and susceptibilities to bias identified in both groups were of the same types and occurred at a similar frequency.

Table S1 describes the characteristics, susceptibilities to bias and limitations of each study reviewed.

### Summary of Information

#### I. Gender

**Figure 2:**
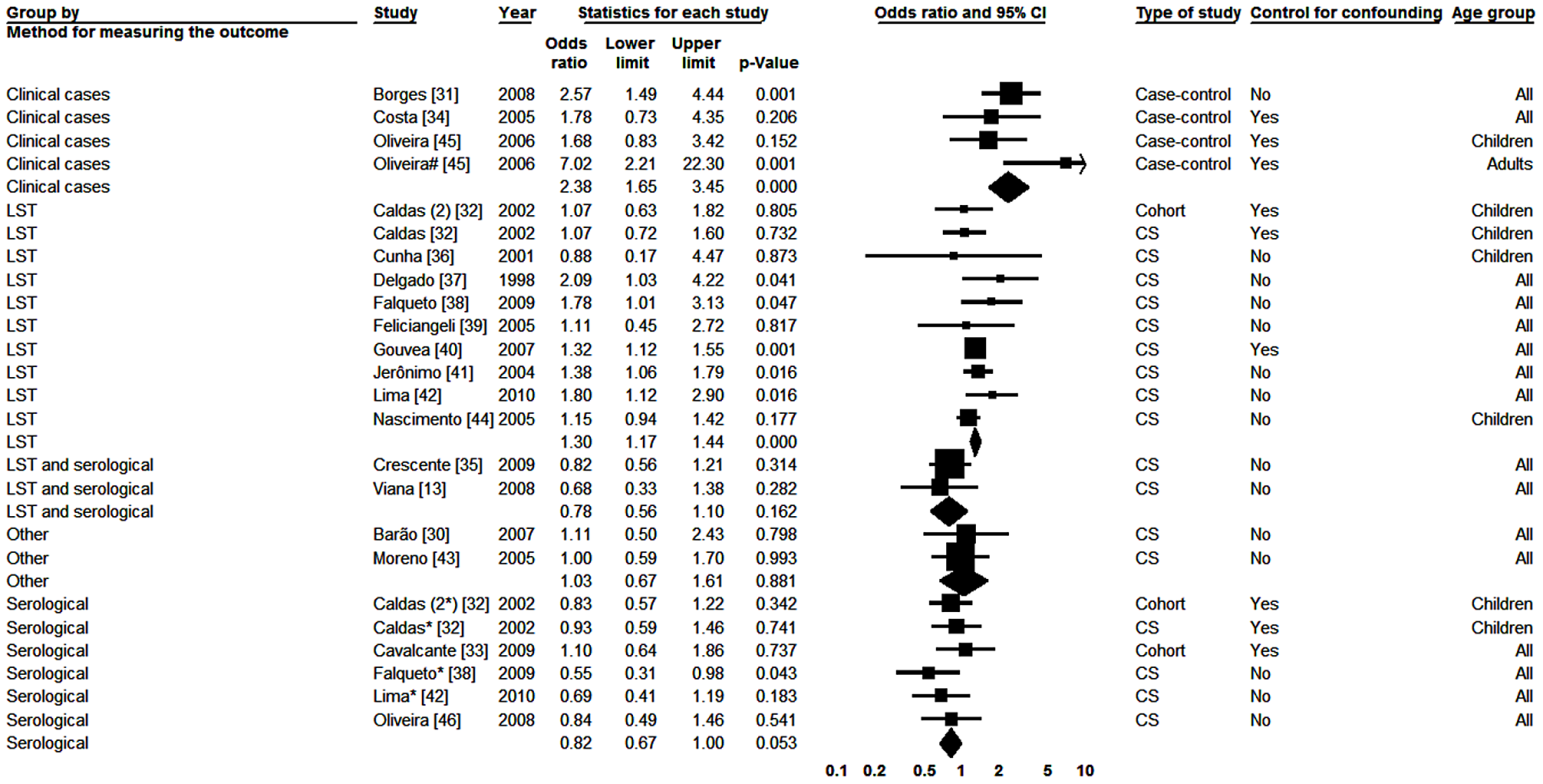
Forest plot for the variable gender: studies divided into subgroups according method for measuring the outcome. CS-Cross-sectional; *Serological result from study with two tests; #Result in adults; (2) Second study from the same publication; LST- Leishmania skin test; Squares represent the weight of each study; Lozenges represent the summary measure of each subgroup; Reference: Females, OR = 1.

Eighteen studies were included [13], [30]–[46]. In one of them, data for adults and children were presented separately [46]. Two types of diagnostic tests were used in four studies, and separate analyses were presented for each of one [32], [38], [42]. In the analysis with no division of studies by subgroups, the effect measure was heterogeneous between studies (p = 0.001) and I^2^ value was 57.73%. When considering the analyses by subgroups of studies, the outcome measurement method was the only grouping characteristic that showed significant differences in the effect measures, significant heterogeneity between subgroups (p<0.001) and lack of heterogeneity within the subgroups (p = 0.625). With the exception of studies where the outcome was the clinical disease, where I^2^ was 37.13%, in all other subgroups the I^2^ value was zero. In the forest plot shown in Figure 2 one notices that in the subgroup of studies in which diagnosis was provided by LST and in studies with clinical cases, the male sex was significantly associated with infection, with odds ratios of 1.30 (1.17–1.44) and 2.38 (1.65–3.45), respectively. As for the others subgroups, although the results were not statistically significant, association was in the opposite direction, with male subjects having a smaller chance of infection.

#### II. Age

**Figure 3:**
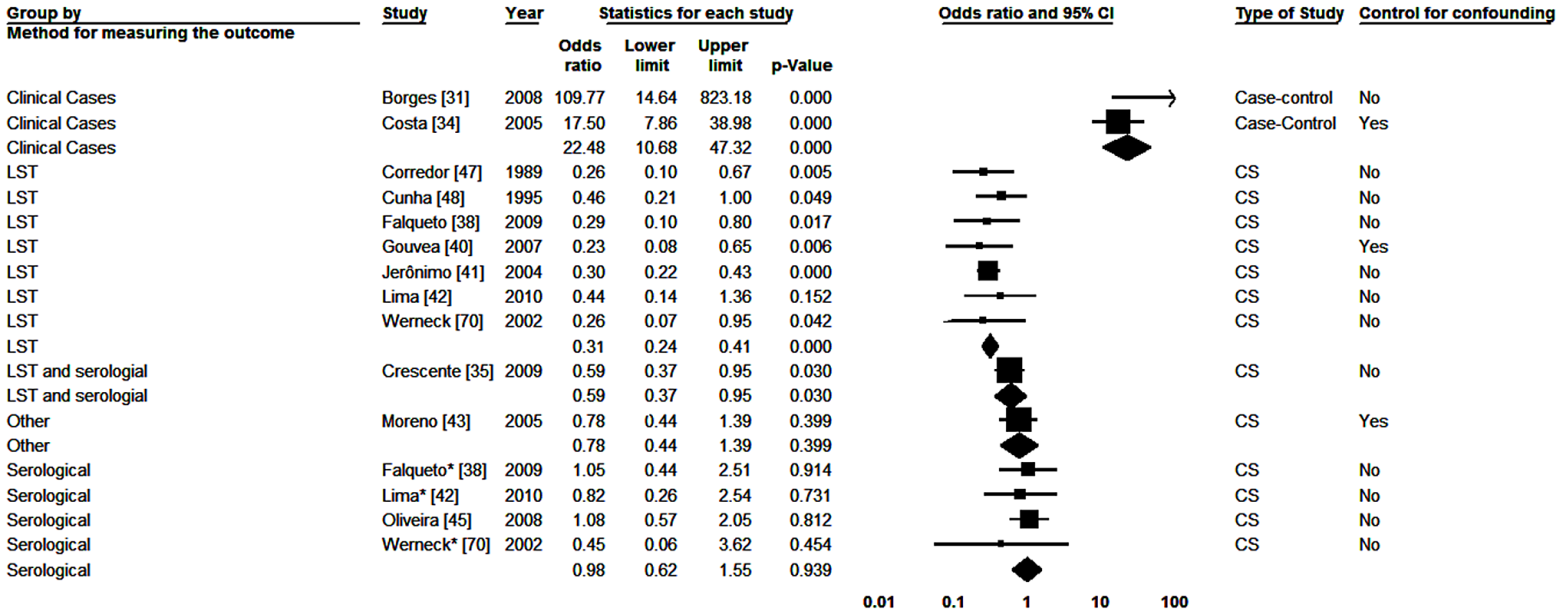
Forest plot for the variable age: studies divided into subgroups according to the method for measuring the outcome. CS-Cross-sectional; *Serological result from study with two tests; #Result in adults; LST- Leishmania skin test; Squares represent the weight of each study; Lozenges represent the summary measure of each subgroup; Reference: Being Over 10 y.o.a., OR = 1.

In order to carry out meta-analysis on age we selected the cut-off point of ten years for comparisons, considering only the fact that this was the one used in most of the primary studies. Twelve studies were analyzed [31], [34], [35], [38], [40], [41], [42], [43], [45], [47], [48], [49]. In three of them [38], [42], [49] two different diagnostic tests were used and analyzed separately. In the overall analysis we detected significant heterogeneity between studies (p<0.001), with I^2^ of 90.34%. In analyses with division of studies by subgroups, statistically significant heterogeneity between subgroups (p<0.001) and not within subgroups (p = 0.867) was found only for the outcome measurement method used. Statistical I^2^ value was 63.71% in the subgroup of studies for which the outcome was the clinical disease and zero for the others. In Figure 3 one notices that both in serological study subgroup and in the LST study subgroup, greater test positivity was found among individuals aged ten years old or more. However, in the last, associations were stronger and statistically significant. Two studies that used other diagnosis procedure showed the same pattern. However, in both studies in which clinical cases was considered the outcome, the direction of the association was the opposite, that is, those with ≤10 years of age with the higher risk.

Three papers reporting results of four studies [30], [33], [50] that compared the occurrence of infection in asymptomatic subjects older and younger than 15 years old (by Kalazar detect dipstick rK39 [30], Elisa and LST [33], [50]) detected the same pattern, that is, older subjects were more likely to be positive.

In the remaining studies that evaluated age, and for which it was not possible to perform a meta-analysis, overall the number of positive subjects also increased with age in studies which performed LST or serological tests [37], [39], [51]. On the other hand, Viana et al. [13] used both tests and did not observe any differences between the average age of positive and negative subjects. For studies which involved children only [32], [36], [44], [52], meta-analysis were not performed since the upper cut-off point was different between studies. In this case, no consistent patterns were identified in the results.

#### III. Malnutrition

**Figure 4:**
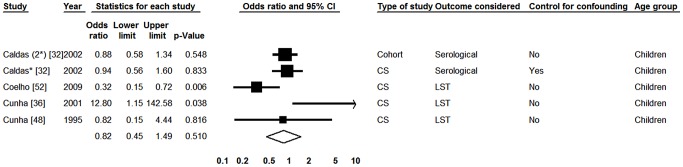
Forest plot: malnutrition. CS-Cross-sectional; *Serological result from study with two tests; (2) According to a study from the same publication; LST-Leishmania skin test; Squares represent the weight of each study; Lozenges represent the summary measure. Reference: Lack of Malnutrition, OR = 1.

In order to evaluate the potential role of malnutrition on the risk of infection by *L. infantum* we selected four papers reporting results of five studies which compared eutrophic children and those with some degree of malnutrition [32], [36], [48], [52]. Figure 4 shows that the presence of malnutrition (although non-significantly) decreased the likelihood of diagnosis of asymptomatic infection. There was significant heterogeneity in the Q test (p = 0.020), with an I^2^ value of 65.88%.

#### IV. Presence of dogs in the household

**Figure 5:**
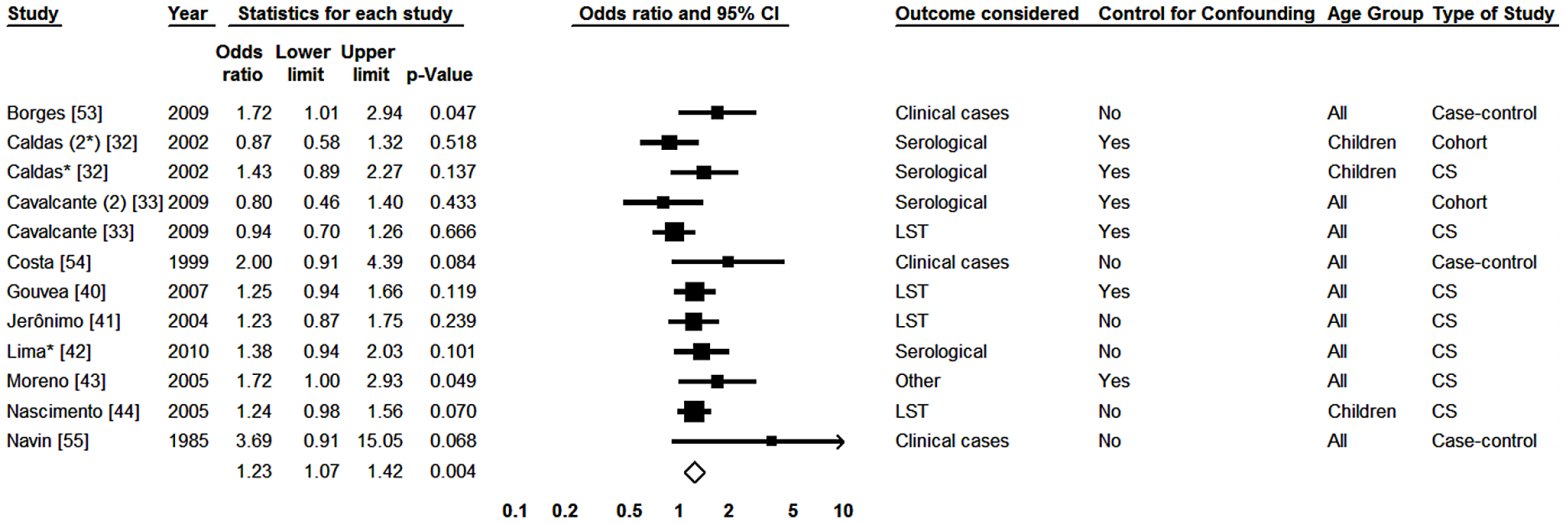
Forest plot for the presence of dogs in the household: studies divided into subgroups according to type of study. CS-Cross-sectional; *Serological result from study with two tests; (2) According to a study from the same publication; LST-Leishmania skin test; Squares represent the weight of each study; Lozenges represent the summary measure; Reference: Not Owning Dogs, OR = 1.

The presence of dogs in the household as a risk factor was evaluated in twelve studies [32], [33], [40]–[44], [53]–[55], and three of them used and provided results using two separate diagnostic methods [32], [33]. In the overall analysis without subgroup stratification, no significant heterogeneity was identified by the Q test (p = 0.152). The I^2^ value was 29.97%. The combined data demonstrated a pattern of increasing likelihood of infection for subjects with dogs in the household (OR = 1.23; 1.07–1.42). However, in both cohort studies analyzed [32], [33] the results were in opposite direction (Figure 5).

#### V. Seropositive dogs

**Figure 6:**
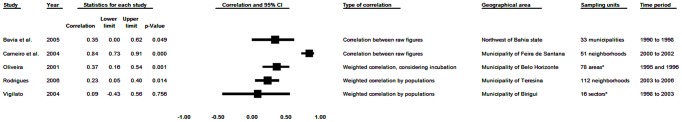
Forest plot with ecological studies that correlated cases in dogs and in humans in a given analyzed land unit. *Terms used in the primary study (for details, see table S1).

For the association between dog seropositivity and the occurrence of VL, most studies used aggregate data, with the exception of one case-control study [53] in which there was a greater history of canine euthanasia in households of cases. In figure 6 we describe five studies providing correlation coefficients between dog seropositivity (assessed by census or sample serosurveys) and the incidence of human VL (based on notified cases) aggregated by geographic units in a given period of time [56]–[60]. In all studies a positive correlation was described. However, the papers of Bavia et al. [56] and of Carneiro et al. [57] have the limitation of analyzing just the number of human and canine cases, not incidence rates or prevalence. In a study conducted in Teresina [61], the multilevel model that provided the best fit to data included the association between the prevalence of dog seropositivity and the incidence of the disease among humans. In Belo Horizonte, in a Bayesian multivariate model for the relative risk of visceral leishmaniasis, the variable with the greatest power for explaining human VL incidence was the prevalence of canine seropositivity [62]. On the other hand, Oliveira and Araújo showed a weak positive correlation between canine prevalence and the incidence rate of VL in humans in a time series study in the municipality of Feira de Santana, Bahia [63]. However this last study has the limitation of analyzing yearly aggregated data for a short period of time (6 years).

#### VI. Chickens/birds/chicken coops

**Figure 7:**
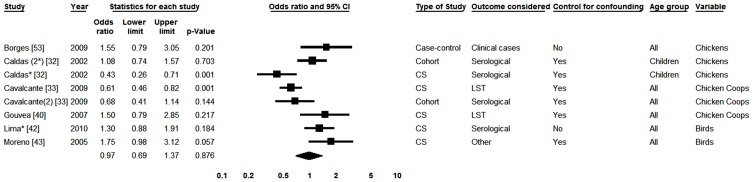
Forest plot for the presence of chickens, birds or chicken coops in the household. CS-Cross-sectional; *Serological result from study with two tests; (2) According to a study from the same publication; LST-Leishmania skin test; Squares represent the weight of each study; Lozenges represent the summary measure; Reference: Not Owning Birds, OR = 1.

Six papers with eight studies included in the review evaluated at least one variable related to the presence of poultry in the peridomestic environment 32,33,40,42,43,53. Information about the presence of chicken coops or birds was only used when there was no information about the presence of chickens in the primary study. Three studies carried out separate analyses for two diagnostic tests [32], [42], [43]. In the overall analysis the I^2^ value was 76.53%. In all subgroups analyzed (including when division was made according to the type of poultry) heterogeneity was maintained. Figure 7 shows a forest plot with the summary measure obtained when all studies were pooled. There was no association between the presence of poultries in the households and the occurrence of infection (OR = 0.97; 0.69–1.37).

#### VII. Other animals

Given the small number of studies or lack of description of results through measures of association, it was not possible to obtain consistent information on the association between the presence of other specific animals and the occurrence of VL [33], [53], [64]. A study conducted by Gouvea et al. [40] analyzed the association between the presence of animals, with the exception of dogs and birds, and the occurrence of infection. They found a high frequency of infection in the presence of animals; however without statistical significance. Other studies analyzed the overall presence of animals and they identified a trend of increased likelihood of infection in households with animals [33], [44]–[46].

#### VIII. VL in relatives and neighbors

**Figure 8:**
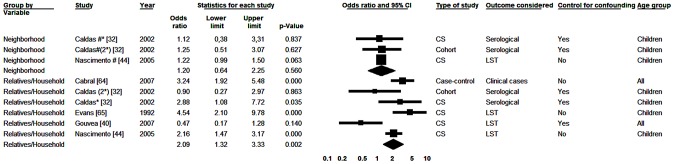
Forest plot: prior cases in relatives or neighbors. CS-Cross-sectional; *Serological result from study with two tests; (2) According to a study from the same publication; LST-Leishmania skin test; #Neighborhood results for studies that also studied relatives; Squares represent the weight of each study; Lozenges represent the summary measure of each subgroup; Reference: Lack of Cases Among Relatives/Neighbors, OR = 1.

We analyzed six studies with respect to prior occurrence of VL in relatives [32], [40], [44], [64], [65] and three for VL in neighbors [32], [44] (two studies analyzed more than one diagnostic method [32]). Figure 8 shows a forest plot for those associations. The summary measures showed greater chance of infection in subjects who referred VL cases among relatives or among neighbors. However, in addition to being stronger (OR = 2.09) the association was only statistically significant for the occurrence of prior cases among relatives.

For the variable “relatives” there was significant heterogeneity of data (p = 0.05 and I^2^ = 70.57%), which did not occur with the variable “neighbors” (I^2^ = 0).

#### IX. Socioeconomic variables

Three studies at the individual level analyzed the association between the existence of a water network connected to public service and the occurrence of infection [34], [40], [44] and showed there was protection for appropriate supply, with an OR of 0.88 (0.69–1.12) in the summary measure. With respect to ecological studies, Braga [66] demonstrated that census tracts where less than 50% of households had piped water suffered greater increase in the incidence of infections. Cerbino-Neto et al. [67] demonstrated that in neighborhoods where there was a lower proportion of piped water, VL incidence was greater. On the other hand, even if the municipality had been the same and only the period of study had changed, Rodrigues [59] identified an opposite pattern to that of previous results [67]. Araújo [62], in turn, did not identify associations between such variables.

With respect to the existence of sewerage system, in three out of four studies which performed analysis at the individual level [33], [34], [40] there was a decrease in the likelihood of infection associated with appropriate sewerage system. The summary odds ratio of data was protective, with a value of 0.78 (0.65–0.95). In ecological studies the association between lack of an adequate sewerage system coverage and an increase in incidence was demonstrated by all four studies analyzed [59], [62], [66], [67], even considering that in only two [66], [67] the results showed statistical significance.

For garbage collection the three studies at the individual level [33], [34], [43] showed that appropriate waste collection was associated with a smaller chance of infection, with an OR of 0.63 (0.49–0.80) in the summary measure. For ecological studies, two [64], [67] of the three studies [59] in which geographic units were used for analysis and in which time was aggregated, showed that the incidence was lower in areas with suitable garbage collection. In another ecological study, Araújo [62] did not identify any association between these variables. Finally, a study conducted in Teresina [66] demonstrated that census tracts where less than 50% of households had appropriate waste collection suffered a greater increase in the incidence of infections.

**Figure 9:**
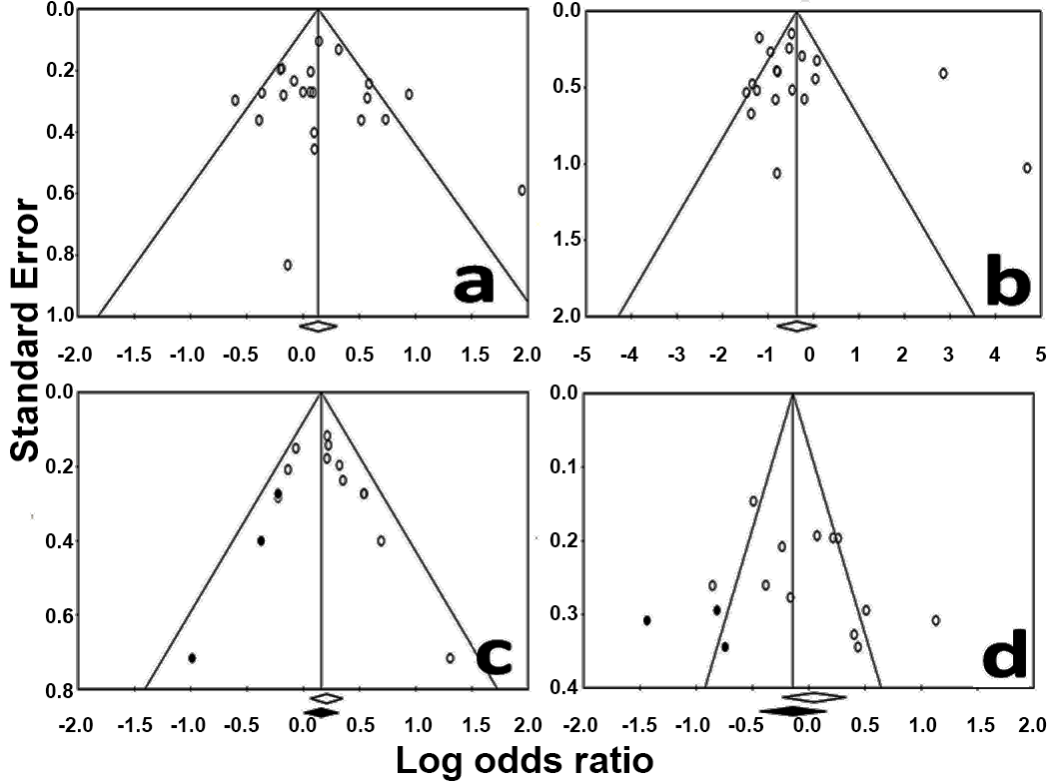
Funnel plot for the variables employed in the detection of publications bias in the review. a-gender; b-age; c-presence of dogs in the household; d-presence of chickens or birds in the household; Dots refer to studies imputed by the Trim and Fill method. Open diamonds represent the original estimate of the Odds ratios while solid diamonds represent Odds rations recomputed by the statistic of Durval and Tweedie's Trim and Fill.

A combination of five studies that compared the existence of inadequate floor surfaces in the household (earth or dirt floor) with another type of floor surface considered appropriate [33], [34], [40], [42], [44] showed that the presence of the latter, albeit with significant heterogeneity in data presented (Q test: p<0.001; I^2^ = 80.51%), was associated with smaller chance of infection, with an OR of 0.50 (0.31–0.80) in the summary measure. In order to analyze the household's finishing, we evaluated eight studies [32]–[34], [40], [43], [44] and the summary measure showed a lower chance of infection when finishing was appropriate (made of bricks and/or concrete, OR = 0.68; 0.47–0.97), albeit also with statistically significant heterogeneity in the Q test (p<0.001) and an I^2^ value of 78.24%. For roof finishing, we analyzed four studies [33], [34], [40], [44] and also verified the pattern of protection for presence of roof tiles, albeit with no statistical significance (OR = 0.85; 0.55–1.30). In the Q test, no statistically significant heterogeneity was identified (p = 0.051), although the I^2^ value was 61.33% (Fig. 9g).

When the subjects' income was directly assessed, with the exception of one study [66], in all others [46], [58], [62], [67], [68] an increase in income was associated with a decrease in the occurrence of the disease.

Seven studies analyzed the relationship between literacy and the occurrence of VL using the subject as a unit for analysis [31], [32], [34], [40], [46], [63]. Despite them all analyzing a similar issue, studies had different cut-off points and definitions so we chose to combine p-values. The value identified with the combination was lower than 0.001. This demonstrates the association between increase in the level of education and decrease in the likelihood of subjects getting infected. In ecological studies that evaluated issues pertaining to education [62], [67], [68], the same pattern was identified. A meta-analysis of p-values was also performed for the number of people living in a household. Four studies with individual units of analysis were combined [34], [40], [43], [46]. Despite the fact that three studies showed an increase in the likelihood of infection in homes that housed more people, there was no statistical significance in the combination (p = 0.072).

The influence of socioeconomic factors in the occurrence of VL was also analyzed by studies that used indicators of social condition including some of the variables analyzed separately in the other studies [46], [69], [70]. All of them demonstrated the association between worse socioeconomic conditions and greater incidence of the disease.

Associations between the occurrence of infection by *L. infantum* and the presence of an automobile or electricity in the household [64] and the subject's knowledge about VL [31] were analyzed in only one study each.

#### X. Environmental and backyard conditions

For the following variables we chose not to summarize data using summary measures of association or p-values, considering the difference between the issues analyzed and data available in primary studies.

The Normalized Difference Vegetation Index (NDVI) was studied in four publications. The NDVI is a vegetation index extracted from remote sensing images which correlates with precipitation, humidity and the presence of green vegetation. Cerbino-Neto et al. [67] identified an interaction of that with the rate of population growth. In areas of dense vegetation and intense population growth there were higher incidence than what would be expected considering the independent effect of those variables. Those authors also demonstrated the association between a higher NDVI value (stratified in units of analysis as greater or smaller than 0.2) and the incidence of VL. In Teresina a greater NDVI mean value in the unit of analysis was associated with an increase in incidence [70]. In a multilevel analysis [61] the highest minimum NDVI value was the best predictor of increase in incidence. Additionally, an interaction with the level of urbanization was also identified. A study conducted in Belo Horizonte [62] analyzed the median NDVI and did not demonstrate any associations. Population growth was specifically associated with an increase in the incidence of VL [66], as well as the greater population density [42], [64]. Finally, studies that analyzed issues pertaining to urbanization showed greater positivity in households closer to the forest [71]; in regions with a smaller proportion of urban area [59], [65], [72]; and in areas with the worst level of urban development [46], [51].

With respect to backyard features, different issues were analyzed. Caldas et al. [32] showed an association between the presence of trees within a 10-meter radius of households and the occurrence of infection. On the other hand, in a study conducted by Moreno et al. [43] people who lived in homes where rubbish was kept in the backyard had lower prevalence. In a study conducted by Gouvea et al. [40], results showed inconsistent patterns; the presence of rubbish without trees or bushes proved to be a factor associated with an increased prevalence of infection, while the presence of trees, bushes and rubbish in the backyard decreased the prevalence of infection. It should be highlighted that results from those studies were not statistically significant. This did not occur, however, when a score was created in which features of the backyard such as lower frequency of cleaning, presence of trees, of waste, of ants and others, were jointly associated with an increase in the chance of infection in multivariate analysis [46].

Associations between rainfall [64] or periods of El Niño [73] and notifications of human visceral leishmaniasis were analyzed in only one study each.

#### XI. Other variables

Two publications [32], [43] detected a greater likelihood of infection for people playing or being outside the home between 18 and 22 hours. The relationship between Phlebotomine sand flies and the incidence of VL was analyzed in three ecological studies only [56], [71], [74] and, as expected, greater abundance of the vector was associated with greater occurrence of the disease.

Other variables also analyzed in literature were: ethnic group [43], degree of restriction of dogs [53], presence of plants in the household [40] and prior blood transfusion [75].

### Publication Bias

The presence of publication bias was analyzed only for four variables since there were not enough publications to discuss its possible influence on the other associations reported. According to the methodology used, no evidence of publication bias was identified for associations regarding gender. In Egger's test, the p-value (one-tailed) was 0.34 and the funnel plot was apparently symmetrical (Figure 9a). The same occurred for age (p = 0.16 in Egger's test) (Figure 9b). For the presence of dogs in the household, however, Egger's test identified the possibility of publication bias (p = 0.042). The analysis of the funnel plot showed that it was slightly asymmetrical (Figure 9c); it was then necessary to impute three studies using the “Durval and Tweedie's Trim and Fill” method to obtain symmetry. The new OR estimated was slightly lower (1.17; 1.01–1.36) but remained statistically significant. Finally, the Egger's test was not significant for the presence of birds (p = 0.06), but the funnel plot was not symmetrical; three studies under “Durval and Tweedie's Trim and Fill” method were imputed in order to obtain symmetry. The recomputed OR was stronger but the association was on the same direction (0.87; 0.65–1.16) and non-significant (Figure 9d).

## Discussion

This review has brought to light several aspects relating to factors associated with *L. infantum* infection in humans in the Americas, as well as limitations in the literature consulted and gaps in the existing knowledge. For the variable gender we verified that in studies with clinical cases and in studies that used LST, male gender was positively associated with VL. However, with serological tests we verified an association in the opposite direction. Despite the fact that only the first two were statistically significant, the observation of the measures of association and the statistically significant difference in the Q test show that the difference between groups was consistent. In five out of six studies with serological tests, LST were also performed, that is, subjects were the same. Previous studies argued that hormonal or immune issues could affect the progress of infection [76], [77], and male subjects would be more likely to become symptomatic. Such assumptions could justify the results of studies with symptomatic cases; however this would not explain the difference identified between types of diagnostic methods for detecting asymptomatic infection. In a study in Teresina, Costa et al. [78] suggested that infection with *L. infantum* more often affects men because they are more exposed to vector bites, since they commonly do not wear shirts due to the high temperatures in that region of Brazil. This can lead to greater exposure of the body surface to the vector. Another postulated explanation for the association between male sex and infection is that men engage more frequently in household tasks or leisure-time activities outdoors, especially after normal working hours, during the peak period of vector activity [40].

For age, it became clear that children are less likely to receive a positive diagnosis of asymptomatic infection. Since LST are indicative of infections that have occurred throughout a subject's entire life [79] and serological tests may remain positive even long after the infection [14], results are within what would be expected. One should also take into account whether epidemiological or behavioral factors could be associated with identified patterns in addition to such characteristics of the tests. Results in opposite directions and with strong odds ratios verified in both case-control studies analyzed reveal that children are more prone to develop clinical disease, despite being less likely to get infected. This fact may be verified when one observes the number of symptomatic cases routinely notified by healthcare services [62].

The presence of dogs in the household was positively associated with VL. Among the variables subjected to meta-analysis, this was the one with the lowest heterogeneity. Besides the fact that the dog is considered the most important reservoir of the infection in urban settings [8], its presence is positively correlated with vector abundance, potentially increasing the risk of transmission [80]. Furthermore, even without the possibility of performing meta-analysis, ecological studies showed consistent evidence that dog seropositivity is associated with human VL. This emphasizes the relevance of dogs at least as a marker of the occurrence of visceral leishmaniasis among humans.

Regarding the presence of chickens and birds in households, although there is evidence that they might have a role in attracting Phlebotomine sand flies [81], [82], according to the available evidence their presence could not be incriminated as a risk factor for VL. Unlike dogs, chickens and birds are not reservoirs of *L. infantum* [83] and they might even divert sandflies attention. Indeed the summary measure obtained shows that a protective role of the presence of such animals cannot be discarded, as already discussed in the literature [84].

With respect to the variables for which no subgroup analysis was performed, malnutrition decreased the likelihood of positive results, possibly due to the immunological techniques employed for the diagnosis. It should be noted, however, that these results do not exclude the role of malnutrition in increasing the risk of developing clinical disease, as already shown in other publications [85], [86]. As for the prior occurrence of VL in relatives or neighbors and its relationship with an increase in positivity, the association was stronger for the first one, the same pattern as shown in a study conducted by D'Oliveira et al. [50]. Since most studies considered as relatives only those who lived in the same household, the focal nature of the disease could explain the data presented. However, genetic factors that may be associated with the infection should not be disregarded. With respect to socioeconomic issues, although the strength of evidence was variable depending on the factor analyzed and the number of studies being small, the set of data presented shows that there is a close relationship between visceral leishmaniasis and poor living conditions, lack of basic urban infrastructure services and low levels of education. Such relationship between low socioeconomic status and the risk of VL could be explained to the extent that poor economic conditions are associated with greater abundance of vectors, with the lack of responsible pet ownership practices and with the location of houses in peripheral areas of the cities where vegetation density is favorable for the presence of vectors and, perhaps, of sylvatic reservoirs [61], [80], [87], [88]. Finally, albeit in a small number of studies, variables relating to features of the environment and vegetation (evaluated through the NDVI) demonstrated that visceral leishmaniasis occurs more frequently in areas where the level of urbanization is lower and where vegetation is more abundant, probably creating adequate habitats for breeding of vector population [89].

Although quality analysis performed in this review also has limitations arising from the fact that there is no single standardized tool recommended to evaluate observational studies [25], in particular regarding specific issues of ecological studies, several limitations and susceptibility to bias could be identified in the studies analyzed. Even though some of those might be considered inevitable, such as the lack of quality of secondary data used in ecological studies, many other limitations and biases could have been avoided if researchers had adopted the correct procedures for selecting participants, measuring variables and analyzing data, and also had they used more standardized, appropriate and transparent ways of reporting their studies. Based on data presented, the following should be considered in future studies: a) strategies to minimize refusals or losses should be strengthened, as well as other possible design-specific biases identified; b) standardized procedures to select participants should be adopted, considering the need for this selection to be random and to take into account a sample size that is appropriate so that the study has enough statistical power; c) combinations of diagnostic tests should be used so that results may be more valid and reliable; d) control for confounding should be performed and interactions between variables should be analyzed; e) analyses with continuous data in their original form should be performed, considering their advantages [90] and in cases where stratifications prove to be necessary, these should be explained and justified in terms of analysis and practical applicability and not only aiming to achieve statistical significance; f) studies should use correct statistical procedures which extend beyond the p-value, also discussing the strength and direction of associations; g) studies should use the STROBE statement so that results are better described, clearer and more standardized.

A number of limitations in the present study deserve mention. Considering that control for confounding was performed in few studies and considering different factors, there may be a large amount of residual confounding in the data summarized in this review. This could mean that the strength of associations might be overestimated. The same overestimation might also have occurred due to the decision to use odds ratio as a measure of association [91], even if it was necessary so that studies which provided only p-values and sample sizes could be used, as well as for the inclusion of results from logistic regression models. Although results from analyses of publication bias showed no consistent evidence of its occurrence, it should be noted that such analyses were limited considering the existing heterogeneity among the various studies results. Furthermore, they could not be performed for most variables. Despite the fact that an extensive search for theses and dissertations was carried out, we did not include studies published in annals of scientific conferences. In addition, for some of the studies included there was significant loss of information on the association for certain variables because non-significant results were not described. Finally, one should consider the low power of some procedures carried out, as well as the limitations imposed by the fact that it was impossible to perform subgroup analyses for most variables. This caused many summary measures to be obtained for heterogeneous data. Such procedures were followed in order to avoid limited and biased analyses based on statistical significance only, such as ‘vote counting’, which we also tried to avoid when discussing the strength and direction of associations even when data allowed theoretical discussions only [28], [92].

The findings of the present review might influence and improve the design of VL control strategies. For instance, the presence of positive dogs might be a useful indicator for monitoring the force of transmission to humans and thus, areas with high canine prevalence or incidence should be prioritized when delivering interventions. Also concerning the reservoir, it should be stressed the importance of policies to promote responsible dog ownership. Additionally, identification of areas at higher risk for prioritizing intervention should take into account the abundance of green vegetation as well as the prior occurrence of VL cases. Since children are more prone to illness and they have the potential to act as information multipliers [93], they might be the focus of attention in health education initiatives. The results related to socioeconomic conditions showed that inadequate urban infrastructure should also be considered a target for interventions against VL. It is of paramount importance to emphasize the need for the formulation of public policies that will improve living conditions and access to education for the population as a whole, since visceral leishmaniasis is just one among other diseases that are rooted into scarcity and poverty [94].

Our work is the first systematic review about factors associated with visceral leishmaniasis in Latin America. Several relevant aspects that help understand the disease dynamics were clarified and new questions were raised. Patterns identified for all variables, as well as the reason for greater chance of infection among adults and greater likelihood of falling ill among children or reasons why the type of diagnostic tests modified the direction of association between gender and asymptomatic infection should be further evaluated. Future studies should investigate whether these patterns are consistent and how subjects' immune response and those characteristics of the tests employed could explain the observed data. With respect to variables about which there is little or inconsistent knowledge, such as subject's level of knowledge about the disease, ethnic group, backyard conditions and the presence of other animals, especially synanthropic and chickens, future studies should build upon the available information. Additionally, the following should also be encouraged: expanding the use of high definition satellite imaging, taking several environmental aspects into account, from backyard features to broader levels, such as those studied by Franke et al. [73], as well as the association with abundance of Phlebotomine sand flies and with the incidence of infections in human beings. Finally, it is also crucial to conduct research in other geographic areas in Brazil and in other Latin American countries where the disease is endemic. More cohort studies are required and the quality of projects, analyses and publications needs to improve dramatically.

## Supporting Information

Table S1 Main features, risks of bias, and limitations in studies selected for systematic review.
(DOC)

Text S1 Search strategies for the PubMed, LILACS, Thesis Databank of CAPES and Google Scholar.
(DOCX)

Text S2 PRISMA checklist. From: Liberati A, Altman DG, Tetzlaff J, Mulrow C, Gotzsche PC, et al. (2009) The PRISMA statement for reporting systematic reviews and meta-analyses of studies that evaluate health care interventions: explanation and elaboration. PLoS Med 6: e1000100. doi:10.1371/journal.pmed1000097.
(DOC)
