# Correction: Factors Associated with Visceral Leishmaniasis in the Americas: A Systematic Review and Meta-Analysis

**DOI:** 10.1371/annotation/83856044-8747-4d93-9a1e-64f16bb60c07

**Published:** 2013-05-03

**Authors:** Vinícius Silva Belo, Guilherme Loureiro Werneck, David Soeiro Barbosa, Taynãna César Simões, Bruno Warlley Leandro Nascimento, Eduardo Sérgio da Silva, Claudio José Struchiner

The published table represents an earlier generation of this file. The most recent generation of this data can be found at the following link: 
