# Supplementary material for: Factors Associated with Visceral Leishmaniasis in the Americas: A Systematic Review and Meta-Analysis
Source: PLoS Negl Trop Dis. 2013 Apr 25;7(4):e2182. doi: 10.1371/journal.pntd.0002182 (PMC3636096; doi:10.1371/journal.pntd.0002182)
Supplement: Table S1 — Main features, risks of bias, and limitations in studies selected for systematic review. (DOC) [file pntd.0002182.s001.doc]

Table 1 Main Features, Risks of Bias and Limitations in Studies Selected for Systematic Review

| **First Author/Ref./Year/Place** | **Population (N)** | **Type of Study and Details** | **Analyzed Variables** | **Outcome** | **Diagnostic Test** | **Analysis and Confounding Control** | **Excluded Variables** | **Main Risks of Bias** | **Main Limitations of Analysis** | **Main Limitations of Description** |
| --- | --- | --- | --- | --- | --- | --- | --- | --- | --- | --- |
|  |  |  |  |  |  |  |  |  |  |  |
| **Araújo / [62] / 2011 / Belo Horizonte – Minas Gerais** | 146 areas covered by the municipali-ty’s Healthcare Service | Ecological / Data from 2007 to 2009 - grouped | Positive dogs / Altitude / Water supply / Sewer / Waste destination / Inhabitants per household / Education / Income / NDVI | Relative risks estimated by Bayesian modeling | Not applicable | Bayesian modeling (Besag for spatial smoothing; Car model for the inclusion of random effects) | None | Secondary data / Possible differential notification across sectors / Possible ecological fallacy | Procedure for entry in the multivariate model was not clearly described | None |
|  |  |  |  |  |  |  |  |  |  |  |
| **Barão / [30] / 2007/ Araçatuba-São Paulo** | 125 subjects from two areas within the municipali-ty | Cross-Sectional | Gender / Age Presence of dogs in households | Infection diagnosis | Kalazar detect Dipstick rK39 | Chi-square test, with no control for confounding | Area of study - local relevance only | There was no description of the process for selecting participants and refusals | Did not explain how variable stratification was defined / Did not provide results for statistical tests | For the presence of dogs the study stated only that this was not significant |
|  |  |  |  |  |  |  |  |  |  |  |
| **Bavia/ [56] 2005/ A sanitary district in northwest Bahia State** | All 33 municipali-ties within the district | Ecological / Data from 1990 to 1998 - grouped | Seropositive dogs / Number of Phlebotomine sand flies | Number of positive cases | Not applicable | Spearman’s correlation, with no control for confounding | Population density and NDVI - it was not possible to understand the analysis | Use of secondary data with no description of how such data were obtained / Unit analyzed was the municipality - potential ecological fallacy / Quality of records may have varied across municipalities / Places where cases occurred not verified | Correlation with the raw number of cases / Did not consider potential spatial dependence | The study stated only whether there was statistical significance or not / Text difficult to understand |
| **Borges / [31] / 2008/ Belo Horizonte – Minas Gerais** | 82 Cases and 160 Controls | Case-control / (Cases contacted through home visits) | Age / Gender / Education / Knowledge about VL | Being identified as a case (identification of cases occurring in 2004 through Sistema de Informações de Agravos de Notificação – Sinan –Ministry of Health of Brasil) | Not applicable | Odds ratios calculation, with no control for confounding | Preventive actions - categories treated as mutually exclusive which should not have been. | No description of how controls were sampled and of refusals in both groups / Possible memory bias / Did not consider subject’s age when analyzing education. | Did not explain how variable stratification was defined / Some associations with  low power | Did not describe the procedure and variables involved in the regression model; consequently only univariate data could be analyzed in the review. |
|  |  |  |  |  |  |  |  |  |  |  |
| **Borges / [53] / 2009 / Belo Horizonte – Minas Gerais** | 82 Cases and 160 Controls | Case-control / (Cases contacted through home visits) | Where the dog stays during the day / where the dog sleeps / Whether dog was positive / History of euthanasia / Presence of: chickens / pigs / opossum / birds / cats / dogs | Being identified as a case (identification of cases occurring in 2004 through Sinan) | Not applicable | Odds ratios calculation, with no control for confounding | Place where subjects got dogs - no epidemiologi-cal relevance / Prior results / Dog’s age / Canine replacement / Presence of stray dogs - impossible to understand the definition or analyses undertaken | No description of how controls were sampled and profile of refusals in both groups / Possible memory bias | Some associations with  low power | Even if this study described the procedure for logistic regression, its results were not described / For some variables it was not possible to obtain measures of association and to use information |
|  |  |  |  |  |  |  |  |  |  |  |
| **Braga / [66] / 2007 / Teresina – Piauí** | 102 neighbor-hoods in the municipali-ty | Ecological / Data from 1996 to 2005 - sorted into three groups #1 1996/1997/ 1998; #2 1999/2000/ 2001/2002; #3 2003/2004 /2005 | Population growth / Water supply / Income / Waste collection / Sewer | Progress of incidence rates by neighborhood classified into two groups: growing-high, but stable OR low, but stable – unstable | Not applicable | Odds ratios calculation, with no control for confounding | None | Secondary data / Possible differential communication across neighborhoods | All variables were dichotomized, which may increase the chance of residual confounding | None |
|  |  |  |  |  |  |  |  |  |  |  |
| **Cabral / [64] / 2007 / Seven Municipali-ties of Natal’s Metropoli-tan Area – Rio Grande do Norte (study 1)** | 143 Cases and 62 Controls | Case-control (identified cases, contacted through home visits and controls who were neighbors of cases) | Other cases in the family / Owning an automobile / Type of floor tiles / Type of walls / Destination of sewer / Water supply / Presence of trees / Presence of animals (overall and individually) / Education | Being identified as a case (data from the State Secretariat of Health of cases that occurred between 1990 and 2006) | Not applicable | Chi-square test, with no control for confounding | All variables pertaining to neighborhood, since controls were neighbors / Water storage - no epidemiologi-cal relevance to VL | Did not describe the control sampling process and refusal / Used a long period for case identification which increases the chance of information bias | Did not explain how variable stratification was defined / Did not discuss the strength of associations / Number of cases much greater than that of controls | Mostly only the p-value was provided without any description of the number of cases and controls in each group; this prevents information from being used. |
|  |  |  |  |  |  |  |  |  |  |  |
| **Cabral / [64] / 2007 / Rio Grande do Norte (study 2)** | 217 municipali-ties within the state | Ecological / Data between 1999 and 2001 - grouped | Rainfall / Electricity / Waste collection / Population density / Human development index (HDI) | Human incidence (per 10,000 inhabitants) | Not applicable | Linear regression, two models were fitted: the first only with HDI and rainfall and the second with the rest | HDI - data analysis bias / School attendance / Education / People who receive government support - impossible to understand | Use of secondary data / Unit analyzed is the municipality - potential ecological fallacy / Quality of records may have varied across municipalities / Places where cases occurred not verified | Did not explain why two regression procedures were followed simultaneously / Normality was not tested / Interactions were not tested | Variables described in methodology did not have the results described / Text difficult to understand |
|  |  |  |  |  |  |  |  |  |  |  |
| **Caldas / [32] / 2002 / Raposa – Maranhão (2 studies)** | 679 children (aged 0 to 5) for the cross-sectional study and 572 for the cohort | Two studies: Initially sectional, then cohort. | Age / Gender / Education / Finishing of house / Malnutrition / Where children play / Presence of animals / VL in neighbors or relatives / Dog / Chicken | Infection diagnosis | Intradermal reaction and Elisa | Cox regression applied to the sectional design, with control for confounding for significant variables in the initial stage (P<0.10) | Phlebotomine sand flies - because information came from subjects’ observation | No information on losses or refusals / Exams were performed on children younger than 6 months. | Did not explain how variable stratification was defined / Did not test interactions and multicollineari-ty was not verified | None |
|  |  |  |  |  |  |  |  |  |  |  |
| **Carneiro / [57] / 2004 / Feira de Santana – Bahia** | 44 neighbor-hoods and seven districts within the municipali-ty | Ecological / Data from 2000 to 2002 - grouped | Seropositive dogs | Number of cases notified | Not applicable | Spearman’s correlation, with no control for confounding | NDVI - impossible to understand the analysis undertaken | Use of secondary data / Possible differential communication across neighborhoods/ Place where cases occurred not verified | Correlation between the raw number of cases, without stratification by population / Did not consider potential spatial dependence | Text difficult to understand. |
|  |  |  |  |  |  |  |  |  |  |  |
| **Cavalcante / [33] / 2009 / Raposa – Maranhão (2 studies)** | 1372 subjects examined at the initial stage and 958 in the second examination | Two studies: Sectional (initial examination) and cohort (comprising two measurements) | Age / Gender / Number of people in the household / Walls / Sewer / Domestic animals (dog; others; dog and others; none) / Chicken coop / Presence of sand flies / Income / Roof / Floor tiles / Waste collection | Infection diagnosis: positive for intradermal reaction - for the sectional study OR negative serology in the first stage and positive in the second testing - for the cohort study | Intradermal reaction (in the first survey) and Elisa (both) | For incidence: logistic regression; for prevalence: Poisson’s regression. Control for confounding for significant variables in the initial stage (P<0.20) | Where subjects live and for how long - local relevance / Presence of breeding sites / Presence of Phlebotomine sand flies - impossible to understand | Large number of refusals and losses without discussing profile / Results from the second serological result may have been influenced by prior Montenegro tests | No details were provided regarding modeling procedures, especially Poisson’s / Interactions or multicollineari-ty were not tested | In the initial stage, only information about significant variables was provided. |
|  |  |  |  |  |  |  |  |  |  |  |
| **Cerbino-Neto / [67] / 2009 / Teresina – Piauí** | 101 neighbor-hoods in the municipali-ty | Ecological / Data from 1991 to 2000 - grouped | Water supply / Waste collection / Sewer / Education / Income / Population growth / NDVI | Average incidence of VL during the period | Not applicable | Pearson’s correlation coefficient with a spatial linear regression model, with control of confounding factors | None | Use of secondary data / Possible differential notification across neighborhoods | None | None |
|  |  |  |  |  |  |  |  |  |  |  |
| **Coelho / [52] / 2009 / Raposa – Maranhão** | 495 children from three districts within the municipali-ty | Cross-Sectional | Age / Malnutrition | Infection diagnosis | Intradermal reaction | Chi-square test, with no control for confounding | None | No description of the profile of refusals | Did not explain how variable stratification was defined / Other variables quantified had only descriptive data presented / Did not analyze the strength and direction of associations | The study reported using Elisa but no results were provided |
|  |  |  |  |  |  |  |  |  |  |  |
| **Corredor / [47] / 1989 / El Callejon (a district in the municipality of Ricaute – Colômbia)** | 84 subjects, all living in the district | Cross-Sectional | Age | Infection diagnosis | Intradermal reaction | No statistical analysis was carried out - measures of association obtained from described information | None | There was no description of the process for selecting participants and refusals / Long study timeframe - two years | Only one variable was analyzed, without any explanation on stratifications performed and with no statistical analysis | None |
|  |  |  |  |  |  |  |  |  |  |  |
| **Corredor / 1999 / [51] / Coyama, Tolima, Colombia** | 454 subjects from two districts in the municipali-ty, in areas of governmen-tal action | Cross-Sectional | Age / Gender / Wall finishing / Floor tile finishing / Presence of animals / Presence of dogs / Number of people in households | Infection diagnosis (in at least one of the tests, with the exception of age which was described for each test) | Intradermal reaction / IFAT / Elisa | Chi-square or Fisher’s exact test, with no control for confounding | Variables relating to control actions / Identification of vector - impossible to understand | Did not discuss the profile of refusals / Study limited to homes under governmental intervention | Did not explain how variable stratification was defined / Did not analyze the strength and direction of associations | Tables showed P-values only without any information on the direction of association / All variables not possibile to use |
|  |  |  |  |  |  |  |  |  |  |  |
| **Costa / [74] / 1990 / Teresina – Piauí** | Each of the semesters in the period of study | Ecological, with data from 1983 to 1986 | Presence of Phlebotomine sand flies in households (proportion with at least one) | Number of human cases notified | Not applicable | Correlation coefficient (did not specify which one) | None | Did not describe the process for collecting Phlebotomine sand flies and how collection sites were chosen / Number and location of investigated households was variable throughout time | Did not describe the correlation analyzed / Households may not have represented a random sample of the municipality / Did not take the disease’s incubation period into account | None |
|  |  |  |  |  |  |  |  |  |  |  |
| **Costa / [54] 1999 / Teresina – Piauí** | 46 cases e 73 controls. | Case-control (cases contacted during home visits and controls contacted within a range of up to 100 meters of cases). | Presence of dogs in the house | Being identified as a case (in the last six months) | Not applicable | Odds ratio calculation, with no control for confounding | None | Did not describe how cases were sampled / No description of the profile of refusals | Analysis was divided into two control groups (20 and 100 meters from cases), with no explanation why / Low statistical power | None |
|  |  |  |  |  |  |  |  |  |  |  |
| **Costa / [34] 2005 / Teresina – Piauí** | 44 cases e 176 controls | Case-control (cases contacted during home visits and controls selected randomly within the municipality) | Age / Gender / Number of people in households / Education / Families living in slums within the census tracts / Type of roof / Type of floor tiles / Finishing / Sewer / Waste collection / Water supply | Being identified as a case (in the last seven months) | Not applicable | Multiple logistic regression with control for confounding for variables of the model | History of migration - local relevance | None | Did not describe the criteria for variable entry in the regression model | None |
|  |  |  |  |  |  |  |  |  |  |  |
| **Crescente / [35] / 2009 / Bacarena – Pará** | 946 subjects from two districts within the municipali-ty | Croos-Sectional | Age / Gender | Infection diagnosis (separately for each method) | Intradermal reaction and IFAT | No statistical procedures were performed | None | No description of the profile of refusals | Did not explain how age stratification was defined / Did not performed statistical procedures | None |
|  |  |  |  |  |  |  |  |  |  |  |
| **Cunha / [48] / 1995 / Monte Gordo – Bahia** | 152 subjects (for age) e 79 children (for nutritional status) | Cross-Sectional | Age / Malnutrition | Infection diagnosis | Intradermal reaction | Chi-square test, with no control for confounding | How long subjects have been living in an area / where subject comes from - local relevance / Presence of a seropositive dog - inconsistent data | There was no description of the process for selecting participants / Large number of refusals with no discussion on their profile | Did not explain how age stratification was defined / Did not analyze the strength and direction of associations | Although Elisa was performed, its results were not described / Text difficult to understand |
|  |  |  |  |  |  |  |  |  |  |  |
| **Cunha / [36] / 2001 / Porteirinha – Minas Gerais** | 26 children receiving care at a primary healthcare unit | Cross-Sectional | Gender / Age / Malnutrition | Infection diagnosis | Intradermal reaction | Fisher’s test (categorical variables) and t test (for age), without control for confounding | None | No refusals were described / Limited sample | Sample was very small / Normality was not tested / Did not analyze the strength and direction of associations | Did not describe the profile of the primary healthcare unit analyzed |
|  |  |  |  |  |  |  |  |  |  |  |
| **Delgado / [37] / 1998 / Village of Guayabita – Venezuela** | 315 subjects living in Vilage | Cross-Sectional | Gender / Age | Infection diagnosis | Intradermal reaction | Chi-square test, with no control for confounding | Occupation - impossible to understand / How long subjects had been living there - local relevance | Did not explain how participants were selected / Did not discuss the profile of refusals | Did not explain how age stratification was defined / Did not analyze the strength and direction of associations | Did not describe results for other diagnosis / No possibility of use for age |
|  |  |  |  |  |  |  |  |  |  |  |
| **D’Oliveira-Júnior / [50] / 1997 / Monte Gordo and Barra do Jacuípe, Bahia** | 135 subjects, relatives or neighbors of previous cases | Cross-Sectional | Age / Being a neighbor or relative | Infection diagnosis (in at least one of the tests) | Intradermal reaction and Elisa | Chi-square test, with no control for confounding factors | None | Did not describe how index cases were obtained / Did not describe refusals / Prevalence may be overestimated | Did not explain how age stratification was defined / Did not analyze the strength and direction of associations | None |
|  |  |  |  |  |  |  |  |  |  |  |
| **Evans / [65] / 1992 / Itapioca and Itapagé – Ceará (2 studies)** | 920 children | Two studies: Cross-Sectional (intradermal reaction) with data from 1989. Cohort (Elisa) in two areas of the municipalities: 6 sample collections between 1987 and 1989 | Household environment (mountain; foothill; savannah; city) / Prior case in the household | Infection diagnosis | Intradermal reaction (for environment) and Elisa (for prior case) | Relative risks calculation, with no control for confounding | Presence of dogs - data had limitations | Inconsistencies in the description of how participants were selected / No description of reasons for refusals / Study did not specify whether there were any losses in the cohort | Cohort maintained positives in the first exam and data analysis did not take into account losses and individual contribution time | Other variables that had been quantified were not analyzed as risk factors / Text and tables difficult to understand |
|  |  |  |  |  |  |  |  |  |  |  |
| **Falqueto / [38] / 2009 / Pancas – Espírito Santo** | Subjects from a rural area: 186 took serological tests and 201 were tested for intradermal reaction | Cross-Sectional / Two surveys are conducted, but data from the first only are analyzed | Age / Gender | Infection diagnosis (separately for each test) | Intradermal reaction and Elisa | Chi-square or Fisher’s exact test, with no control for confounding | None | Large number of refusals with no discussion about their profile / Limited sample | Did not explain how age stratification was defined / Did not analyze the strength and direction of associations | Did not explain why data from the second survey were not analyzed |
|  |  |  |  |  |  |  |  |  |  |  |
| **Feliciangeli/ [39] / 2005 / Curarigua, Lara, Venezuela** | Subjects from a rural community: 79 tested for intradermal reaction; 80 for IFAT | Cross-Sectional: despite two surveys being conducted, only the second was selected for literature review (for being more recent) | Age / Gender | Infection diagnosis (separately for each test) | Intradermal reaction / IFAT / Dipstick test, based on rK39 recombinant | Chi -square test and odds ratio calculation, with no control for confounding | Occupation / Presence of positive dogs - impossible to understand analyses undertaken | Large number of refusals with no discussion on their profile | Did not explain how age stratification was defined | Lack of information on age |
|  |  |  |  |  |  |  |  |  |  |  |
| **Feliciangeli / [71] / 2006 / Curarigua, Lara – Venezuela** | 79 subjects from a rural community living in 18 households | Cross-Sectional / | Distance from home to the forest / Abundance of Phlebotomine sand flies | Infection diagnosis | Intradermal reaction | Odds ratio calculation, with no control for confounding | None | There was no discussion over the profile of refusals / Phlebotomine sand flies had been captured four years prior to analyzed cases | The number of people in each household was not taken into account; this caused each home with cases to have the same weight in analysis (for the variable distance) | No tables were provided with measures of association / Text difficult to understand |
|  |  |  |  |  |  |  |  |  |  |  |
| **Franke / [73] / 2002 / Bahia - Brazil** | State of Bahia, Brazil | Ecological / El Niño data from 1980 to 1998 and data from cases that occurred from 1985 to 1999. | El Niño data - Pacific surface temperature (average index) | Annual incidence of VL (per 10,000 inhabitants) - relationship with data from previous year - | Not applicable | Cross-correlation function and linear correlation, with no control for confounding | None | Secondary data / Quality of information may have varied across analyzed data | None | None |
|  |  |  |  |  |  |  |  |  |  |  |
|  |  |  |  |  |  |  |  |  |  |  |
| **Gouvea / [40] / 2007 / Teresina – Piauí** | 1106 subjects from seven neighbor-hoods in the municipali-ty | Cross-Sectional | Gender / Age / Education / People in the household / History of VL in the household / Roof tiles / Roof / Walls / Water supply / Sewer / Dog / Other animals / Presence of a pig pen / cattle pen / chicken coop / Backyard / Plants in the household | Infection diagnosis | Intradermal reaction | Poisson’s regression model with robust variance, control for confounding for significant variables in the univariate analysis (P<0.2) | Subject has lived outside of Teresina and how long they have been in that household - local relevance only | Refusal, albeit small, was greater among children and they were replaced by their parents | Did not explain how variable stratification was defined / Did not test interactions and multicollineari-ty was not verified | None |
|  |  |  |  |  |  |  |  |  |  |  |
| **Jerônimo / [41] / 2004 / Natal – Rio Grande do Norte** | 950 subjects, relatives or neighbors of previous cases | Cross-Sectional | Presence of dogs in the house / Presence of other animals in the house | Infection diagnosis | Intradermal reaction | Chi-square test, with no control for confounding factors | None | Large number of refusals with no discussion on their profile / Prevalence may be overestimated | No statistical test was used to analyze the presence of dogs / Few homes had no dogs, weakening comparison between homes / Did not analyze the strength and direction of associations | Text difficult to understand / No possibility of use for presence of animals, since only the p-value (without the direction of the association) is provided |
|  |  |  |  |  |  |  |  |  |  |  |
| **Lima / [42] / 2010 / Parnamirim – Rio Grande do Norte** | 345 subjects from three neighbor-hoods in the municipali-ty | Cross-Sectional | Population density / Age / Gender / Water supply / Trees within a 10 meter radius / Presence of dogs / cats / birds / donkeys and horses / dogs in the neighborhood / Street paving / Type of floor tiles | Infection diagnosis (separately for each technique) | Intradermal reaction and Elisa | Chi-square test, with no control for confounding | Family income / Number of people in the household / Waste collection / Type of vegetation / soil / in the area / Type of wall used in homes / Destination of water or sewer - impossible to understand the definition or stratifications | Variables associated to neighborhood refer to observations made by locals and may suffer from low validity / No refusals were mentioned | Did not explain how variable stratification was defined / Did not analyze the strength and direction of associations | For most variables there were no descriptive data, however the p-value and the direction of association were provided |
|  |  |  |  |  |  |  |  |  |  |  |
| **Luz / [75] / 2007 / Natal – Rio Grande do Norte** | 151 subjects undergoing dialysis treatment | Sectional / Despite the study covering other populations, for these no factors associated with the infection were assessed | Prior blood donation | Infection diagnosis | Elisa | Not described | Indicator about contact with infection - impossible to understand | There was no description of the process for selecting participants and refusals / Limited sample / Possible information bias / Association difficult to identify due to the nature of the study | Statistical procedure not described / Did not analyze the strength or direction of the association | Text difficult to understand, with different populations, and association was assessed for only one of them / No descriptive information; however p-values and direction were provided. |
|  |  |  |  |  |  |  |  |  |  |  |
| **Miranda / [69] / 2008 / Pernambu-co** | 184 municipali-ties within the state, in addition to the island of Fernando de Noronha | Ecological / Data between 2000 and 2006 - grouped | Indicator of social need (urbanization; urban population; education; occupation; family income; sewer; water supply; destination of waste; people per household) | Incidence of the disease during that period (per 100,000 inhabitants) | Not applicable | Relative risk (municipalities grouped by quartiles, incidence in the worst level divided by that in the best level) / Correlation coefficient - linear; log-linear and multiplicative | None | Unit analyzed was the municipality - potential ecological fallacy/ Quality of records may have varied / There was great loss of information on variables | Did not describe why only municipalities with cases were analyzed - in correlation / Did not test data normality / Did not consider a possible spatial dependence | None |
|  |  |  |  |  |  |  |  |  |  |  |
| **Moreno / [43] / 2005 / Sabará – Minas Gerais** | Of 1604 firstly examined subjects (from a neighborhood in the municipali-ty), 102 seropositive (al in the Elisa or IFAT and 50% of the strip-test) and 124 seronegati-ves were analyzed in the review | Cross-Sectional | Age / Gender / Ethnic group / People per room in the household / Covering / Destination of waste / Presence of a kennel / chicken coop / trees / leaves / Being away from home between 18:00 and 22:00 hours/ Being familiar with the vector / Presence of dogs / birds (in cages) / chickens / dogs with VL | Presence of infection (study analyzed two models and we only kept in the systematic review the one that considered positivity only in hybridization, regardless of other tests - since only in this model the positive subject in hybridization would not be excluded) | IFAT; Elisa; Rapid test L. donovani-TRALd for the initial group / IFAT, Elisa PCRand  Hybridi-zation in the group analyzed in this review. | Logistic regression model, with control for confounding for significant variables in the univariate analysis (P<0.20) | Place of birth - local relevance / Dogs with short hair - there was “presence of dogs” already / Alcohol intake - no epidemiologi-cal relevance | Did not describe how negatives reexamined were selected / Did not describe refusals in both stages | Did not explain how variable stratification was defined / Did not test interactions and multicollineari-ty was not verified | Did not justify the selection and analysis processes followed / Did not describe results from non-significant variables |
|  |  |  |  |  |  |  |  |  |  |  |
| **Nascimento / [44] / 2005 / São José de Ribamar – Maranhão** | 1520 subjects (under 15 years old) from a settlement within the municipali-ty | Cross-Sectional | Gender / Age / VL in the family / Roof / Wall / Floor tiles / Water supply / Presence of animals / of domestic dogs | Infection diagnosis | Intradermal reaction - serology was also performed; not clear what population was examined, hence its exclusion | Chi-square test, with no control for confounding | Prior visceral leishmaniasis - this would be associated with a positive intradermal reaction / Where subjects came from - local relevance / Presence of door or window in the household - predominance within the population | There was no description of the process for selecting participants and refusals | Did not explain how variable stratification was defined / Did not analyze the strength and direction of associations | Analyses of associations with Elisa had to be excluded due to inconsistency and to the fact that it was impossible to understand data provided |
|  |  |  |  |  |  |  |  |  |  |  |
| **Navin / [55] / 1985 / Tegucigalpa – Honduras** | 35 cases and 35 controls. | Case-control (identified cases, contacted through home visits; neighboring controls - paired by age) | Features of homes (thin wood or adobe-stones) / What subjects did at twilight (in or outside the house) / Presence of dogs / Number of dogs / Presence of pigs / goats/ chickens | Being identified as a case (occurred between 1970 and 1983) | Not applicable | Chi-square test, with no control for confounding | Being breastfed | Period is too long to consider the inclusion of cases / When no control was identified the case was excluded | Low statistical power / Overpairing may have occurred / Did not analyze the strength and direction of associations | No additional information was provided for variables that were not statistically significant, which made it impossible to use them |
|  |  |  |  |  |  |  |  |  |  |  |
| **Oliveira / [45] / 2008 / Três Lagoas – Mato Grosso do Sul** | 220 subjects living in households where other symptom-matic cases had already occurred | Cross-Sectional | Gender / Age | Infection diagnosis (in at least one test) | IFAT and Elisa | Chi-square test, with no control for confounding | Other variables in the study - participants came from households where other cases had occurred; this makes comparisons other than at the individual level invalid | No discussion about the profile of refusals / Prevalence may be overestimated | Did not explain how variable stratification was defined / Did not analyze the strength and direction of associations | Did not describe results for a few variables |
|  |  |  |  |  |  |  |  |  |  |  |
| **Oliveira / [58] / 2001 / Belo Horizonte – Minas Gerais** | Areas covered by the Municipal Healthcare Service (78, 67 and 60, respectively in the first, second and third periods of study) | Ecological / Data from 1994 to 1997; study analyzed the association between canine prevalence 6 months prior, in relation to human incidence the following year, totaling three periods and analyses | Prevalence of infection in dogs (6 months prior) | Incidence of human cases (smoothing by a Bayesian method) | Not applicable | Linear regression (three simple linear regressions were performed separately, one for each period), with no control for confounding | None | Secondary data / Possible differential communication across neighborhoods/  Methods of the public control program varied throughout the years, thus areas decreased gradually | None | None |
|  |  |  |  |  |  |  |  |  |  |  |
| **Oliveira / [46] / 2006 / Belo Horizonte – Minas Gerais** | 109 cases and 166 controls | Case-control (cases identified through contact during home visits; controls from two groups: neighbors and subjects selected from hospital admission lists where cases had been treated). | Gender / Income / Education / Type of housing / Presence of animals / Urbanization (good conditions; poor conditions; area of transition or rural) / Scores: features of homes / features of area surrounding homes / Score related to animals in the surrounding area | Being identified as a case (identified through checks with the Municipal Healthcare Service, responsible for distributing medications) - cases occurring between July 1999 and December 2000. | Not applicable | Logistic regression, with control for confounding for significant variables in the univariate analyses (P<0.25) | Marital status - no epidemiologi-cal relevance / Age - controls were already paired according to this variable | There may have been a memory bias / Number of hospital controls was smaller, since there was no access to patient lists in four hospitals | Did not explain how variable stratification was defined / Did not test multicollineari-ty | None |
|  |  |  |  |  |  |  |  |  |  |  |
| **Oliveira / [63] / 2003 / Feira de Santana – Bahia** | Each year in the period of study | Ecological / Data from 1995 to 2000 | Positive dogs | Incidence of human cases (per 100,000 inhabitants) | Not applicable | Pearson’s correlation coefficient with control for confounding for government control measures | Variables relating to VL control | Use of secondary data / Healthcare Service actions to measure canine prevalence changed throughout time | Number of years examined was small / Did not test normality / There was a small variation in canine prevalence, which may have caused correlation to become unfeasible / Did not take the disease’s incubation period into account / Did not test interactions or multicollineari-ty / Did not consider data’s possible time structure | Did not mention the number of animals examined or where such examinations were undertaken |
|  |  |  |  |  |  |  |  |  |  |  |
| **Rodrigues / [59] / 2006 / Teresina – Piauí** | 112 neighbor-hoods in the municipali-ty | Ecological / Data from 2003 to 2006 – grouped | Water supply / Waste collection / Sewer / Income / Proportion of urban area / Proportion of green area / Abundance of vector / Positive dogs | Incidence of human cases (per 100,000 inhabitants) | Not applicable | Linear regression, with control for confounding | None | Secondary data / Possible differential notification across neighborhoods/  Phlebotomine sand fly collection and dog examinations performed by the healthcare service were irregular and limited / Did not verify where cases occurred | Did not consider possible spatial dependence / Did not test interactions | Tables were provided with codes for variables, which made them difficult to interpret |
|  |  |  |  |  |  |  |  |  |  |  |
| **Souza / [68] / 2010 / Bauru-São Paulo** | Census tracts of the municipali-ty grouped as: with or without cases | Ecological / Data from June 2003 to October 2008 – grouped | Schooling / Income | Occurrence of cases within the census tract (yes or no) | Not applicable | Mann-Whitney test (comparing variable medians in areas with and without cases), no control for confounding | Private households - houses / Water supply / Waste collection - the median of variables was 100% | Secondary data with no explanation on how they were obtained / Possible differential communication across neighborhoods/ Place where cases occurred not verified | Possibility of ecological fallacy may have been greater due to how analysis was conducted / Number of cases in sectors was not taken into account / Did not consider possible spatial dependence | None |
|  |  |  |  |  |  |  |  |  |  |  |
| **Thompson / [72] / 2002 / Canindé – Ceará** | 73 areas  measuring 2kmx2km (4km^2^) within the municipali-ty; only those inhabited were included | Ecological / Data from 1981 to 1997; incidence was calculated based on individual-time, presuming the existence of a cohort | Rainfall index / Areas: Foothill (base of a mountain rage) or plain | Annual case incidence; different models were used to estimate population figures | Not applicable | Poisson’s regression, with control for confounding | None | Secondary data / Long timeframe with no information on how data were obtained / Small units of analysis, there may be classification errors / Possible differential communication | There may have been bias due to assumptions made so the analysis could be based on a cohort / Did not consider possible spatial dependence / Did not test interactions and multicollineari-ty | None |
|  |  |  |  |  |  |  |  |  |  |  |
| **Viana / [13] / 2008/ Belo Horizonte – Minas Gerais** | 138 subjects, neighbors of 25 cases | Cross-Sectional (two surveys) | Age / Gender | Infection diagnosis (positive in at least one of the tests, in at least one of the surveys) | Intradermal reaction / IFAT / Elisa | Chi-square (for Gender) and Mann-Whitney test (for age). | None | No discussion about the profile of refusals and losses among surveys / Prevalence may be overestimated (two surveys, three tests) | Did not analyze incidence, despite two sectional studies being carried out / Did not analyze the strength and direction of associations | Did not provide variances |
|  |  |  |  |  |  |  |  |  |  |  |
| **Vigilato / [60] / 2007 / Birigui – São Paulo** | 16 sectors within the municipali-ty (according to the criterion of the National Program to Fight Dengue) | Ecological / Data between 1998 and 2003 - grouped | Prevalence of canine infection | Incidence of human cases (per 100,000 inhabitants) | Not applicable | Spearman’s correlation, with no control for confounding | None | Secondary data / Possible differential communication across sectors/ Possible ecological fallacy / Place where cases occurred not verified / Actions changed throughout the years | Limited correlation since five census tracts did not present any human cases and in others variability was minimal / Only 39 dogs were examined in the first period / Small number of sectors / Did not consider possible spatial dependence / Did not test normality | None |
|  |  |  |  |  |  |  |  |  |  |  |
| **Werneck / [70] / 2002/ Teresina – Piauí** | 430 census tracts within the municipali-ty | Ecological / Data from 1993 to 1996 - grouped | Slum area - based on socioeconomic indicators / NDVI | Incidence of human cases | Not applicable | Spatial regression with spherical covariance, with control for confounding | None | Secondary data / Possible differential communication across sectors | Did not test interactions | None |
|  |  |  |  |  |  |  |  |  |  |  |
| **Werneck / [49] / 2002 / Teresina – Piauí** | 200 subjects from 200 randomly selected homes | Cross-Sectional | Age | Detection of infection (separately according to technique) | IFAT / Elisa / Intradermal reaction | Cuzick Trend Test, with no control for confounding | None | Relevant number of losses for Montenegro’s reaction | Did not explain how age stratification was defined | None |
|  |  |  |  |  |  |  |  |  |  |  |
| **Werneck / [61] / 2007 / Teresina – Piauí** | 430 census tracts and 39 districts (each district with at least three census tracts) | Ecological / Data from human cases from 1993 to 1996 and from canine cases from 1987 to 1994 grouped as follows: (1987–1988, 1989–1990,  1991–1992, & 1993–1994) | Score considering: water supply; sewer; waste collection; education; income; number of people in the household; slums / NDVI / Urbanization / Canine prevalence | Incidence of human infection | Not applicable | Several models were adjusted with a multi-level approach; the study described the the best fitted models | None | Secondary data / Possible differential communication across units / Long period for canine data, there may have been changes to procedures used by healthcare services to obtain data | None | None |
