# Supplementary material for: Factors Associated with Visceral Leishmaniasis in the Americas: A Systematic Review and Meta-Analysis
Source: PLoS Negl Trop Dis. 2013 Apr 25;7(4):e2182. doi: 10.1371/journal.pntd.0002182 (PMC3636096; doi:10.1371/journal.pntd.0002182)
Supplement: Text S1 — Search strategies for the PubMed, LILACS, Thesis Databank of CAPES and Google Scholar. (DOCX) [file pntd.0002182.s002.docx]

**Text S1:** Search strategies for the PubMed, LILACS, Thesis Databank of CAPES and Google Scholar

| **1ª. Pubmed / Medline**: (Leishmaniasis, Visceral OR Leishmania infantum) AND (risk factors OR associated factors OR epidemiological studies OR immunology OR epidemiology).  2ª. **Lilacs:** (visceral leishmaniasis OR leishmaniose visceral AND risk factors OR immunology)  **3ª Thesis Databank – CAPES*:** (Leishmaniose visceral)  **4ª Google Scholar**: Leishmaniose visceral e fatores de risco |
| --- |

*CAPES databank is a collection of theses and dissertations published by Brazilian academic institutions**(.)**
